# Editorial: Prenatal diagnosis and follow-up of children with CNS abnormalities diagnosed in uterus

**DOI:** 10.3389/fneur.2025.1652099

**Published:** 2025-07-08

**Authors:** Barbara Scelsa, Jeanine M. M. van Klink, Chiara Doneda, Mariano Matteo Lanna

**Affiliations:** ^1^Department of Pediatric Neurology, Buzzi Children's Hospital, Milan, Italy; ^2^Department of Neonatology, Leiden University Medical Center, Leiden, Netherlands; ^3^Department of Pediatric Radiology and Neuroradiology, Buzzi Children's Hospital, Milan, Italy; ^4^Fetal Therapy Unit “U Nicolini”, Buzzi Children's Hospital, Milan, Italy

**Keywords:** fetal neurology, fetal MRI, brain malformation, central nervous system (CNS), fetal counseling, neurosonography of fetus

Central nervous system (CNS) abnormalities in the fetus can result from a broad spectrum of maternal, fetal, and placental disorders. Over the past decade, the field of fetal neurology has emerged as a distinct and rapidly evolving subspecialty within pediatric neurology. This growth reflects not only the increasing complexity of prenatal diagnosis but also the recognition that fetal neurological care requires specific training, interdisciplinary collaboration, and a reframing of how we understand early brain development (1). Fetal neurology is no longer simply an extension of pediatric neurology—it is a domain with its own challenges and expertise. Counseling families following prenatal diagnosis of a CNS anomaly is intrinsically multidisciplinary. It typically involves maternal-fetal medicine specialists, neuroradiologists, neurologists, geneticists, psychologists, and pediatric subspecialists, especially when extracerebral malformations are present (2). The multidisciplinary team is asked to interpret and contextualize findings that range from well-characterized malformations with defined prognoses to subtle or ambiguous anomalies that raise more questions than answers. Many fetal CNS findings fall into gray zones—isolated anomalies that may represent benign variants of normal development, or more complex neurodevelopmental disorders. This uncertainty places a significant burden on clinicians and families, underscoring the need for better data, clearer guidelines, and communication strategies (3).

Recent advances in neuroimaging have significantly improved the detection and characterization of brain malformations during gestation (4). High-resolution neurosonography and fetal MRI are increasingly being performed as early as the second trimester, with the belief that earlier diagnosis could open the door to intrauterine therapies. Quantitative approaches and functional MRI techniques are emerging tools that may enhance our ability to assess fetal brain development *in vivo* (4, 5). Alongside imaging, the incorporation of genetic testing—particularly whole-exome and genome sequencing—has improved diagnostic yield (6). These tools are now often available within timeframes compatible with pregnancy management, although incidental findings and ethical dilemmas remain significant obstacles in the counseling process.

This editorial introduces a collection of articles featured in this Research Topic, illustrating the extent and complexity of this interdisciplinary field.

Mark Scher's review offers an impactful overview of the maternal–placental–fetal triad and introduces the concept of the neuroexposome—the sum of internal and external exposures that can shape brain development throughout the lifespan (7). Scher argues that child neurologists must expand their expertise to include not only fetal and neonatal neurology, but also the “science of uncertainty”, embracing the undefined nature of early diagnosis and the complex interplay of factors influencing outcome. Health inequity and global disparities in access to prenatal diagnostics and care are also considered in his analysis, suggesting that global changes are required to ensure equitable care for all families. Three illustrative case vignettes demonstrate the need for interdisciplinary communication and the risks of fragmented or siloed care (Scher).

The article by Roychoudhury et al. explores another emerging frontier: the role of environmental stressors in shaping neurodevelopmental outcomes in preterm infants. Their study draws intriguing connections between the timing of conception and long-term neurodevelopment, suggesting that the month of conception may result in environmental exposures with potential impact on the fetus. This hypothesis could lay the groundwork for new strategies to mitigate modifiable prenatal stressors and improve outcomes in preterm populations (Roychoudhury et al.).

Nutritional influences on brain development are explored by Hu et al. in a meta-analysis evaluating the role of prenatal and early postnatal supplementation of docosahexaenoic acid (DHA). Malnutrition and micronutrient deficiencies—often overlooked in developed countries—remain key contributors to altered neurodevelopment, not only in infancy but in the intrauterine environment as well. While the benefits of DHA are increasingly supported, the review calls for more rigorous, placebo-controlled trials to assess the efficacy of DHA (Hu et al.).

An additional level of complexity is addressed in the article by Bersani et al., which focuses on genetic predispositions to intracranial hemorrhage. Variants in genes such as COL4A1 have been implicated in cases of perinatal intracranial bleeding, and many of these events likely originate *in utero*. As our understanding of the genetic architecture of fetal brain vulnerability evolves, so does the need to explore how genetic factors interact with the external environment to shape individual trajectories. There remains much to learn about how stressors—both internal and external—converge to influence the timing and severity of neurological injury (Bersani et al.).

Looking ahead, the landscape of child neurology is ready to undergo a profound transformation. Earlier and more precise diagnoses during pregnancy, paired with emerging therapeutic strategies—including stem cell therapies, *in utero* surgical interventions, and enzyme/gene therapies—will redefine what is possible. As interventions shift earlier in gestation, postnatal management will also need to adapt. Children with conditions diagnosed and potentially treated *in utero* may follow neurodevelopmental trajectories markedly different from historical cohorts (8).

A recent survey highlights the heterogeneity in clinical practice, reinforcing the urgent need for standardized guidelines, shared protocols, and national or international registries to collect meaningful long-term outcome data in fetal neurology. Notably, these advances require a fundamental shift in medical training. Pediatric neurologists must become familiar with fetal imaging, genetics, intrauterine interventions, and the ethics of prenatal counseling (9). Formal training programs in fetal neurology—structured, interdisciplinary, and evidence-based—are no longer optional but essential.

In conclusion, fetal neurology represents one of the most dynamic and promising frontiers in our field. Its development will not only improve early diagnosis and counseling but also pave the way for timely, targeted therapies that begin before birth. The future of pediatric neurology begins *in utero*. However, despite major advances in prenatal imaging and genetics, our understanding of fetal CNS abnormalities remains incomplete. One of the research gaps in the literature is the predominance of small, heterogeneous cohorts, often with short-term, non-standardized follow-up. Given the rarity of many isolated malformations, the creation of large, single-center cohorts remains a challenge. Collaborative efforts, including multicenter registries and longitudinal studies, are urgently needed to generate reliable prognostic data and inform evidence-based counseling.
